# Effects of Glycemic Index and Cereal Fiber on Postprandial Endothelial Function, Glycemia, and Insulinemia in Healthy Adults

**DOI:** 10.3390/nu11102387

**Published:** 2019-10-06

**Authors:** Glenn A. Gaesser, Jessica Rodriguez, James T. Patrie, Corrie M. Whisner, Siddhartha S. Angadi

**Affiliations:** 1 College of Health Solutions, Arizona State University, Phoenix, AZ 85004, USA; cwhisner@asu.edu (C.M.W.); sangadi@asu.edu (S.S.A.); 2 Soriant Solutions, Roswell, GA 30075, USA; jrvmsv@gmail.com; 3 Public Health Sciences, University of Virginia, Charlottesville, VA 22903, USA; jp4h@virginia.edu

**Keywords:** flow-mediated dilation, glucose, insulin, insoluble fiber, vascular, cardiovascular disease

## Abstract

Both glycemic index and dietary fiber are associated with cardiovascular disease risk, which may be related in part to postprandial vascular effects. We examined the effects of both glycemic index (GI) and dietary (mainly cereal) fiber on postprandial endothelial function. Eleven adults (5 men; 6 women; age = 42.4 ± 16.1 years; weight = 70.5 ± 10.7 kg; height = 173.7 ± 8.7 cm) consumed four different breakfast meals on separate, randomized occasions: High-Fiber, Low-GI (HF-LGI: Fiber = 20.4 g; GI = 44); Low-Fiber, Low-GI (LF-LGI: Fiber = 4.3 g; GI = 43); Low-Fiber, High-GI (LF-HGI: Fiber = 3.6 g; GI = 70); High-Fiber, High-GI (HF-HGI: Fiber = 20.3 g; GI = 71). Meals were equal in total kcal (~600) and macronutrient composition (~90 g digestible carbohydrate; ~21 g protein; ~15 g fat). The HF-LGI meal resulted in a significant increase in flow-mediated dilation (FMD) 4 h after meal ingestion (7.8% ± 5.9% to 13.2% ± 5.5%; *p* = 0.02). FMD was not changed after the other meals. Regardless of fiber content, low-GI meals resulted in ~9% lower 4-h glucose area under curve (AUC) (*p* < 0.05). The HF-LGI meal produced the lowest 4-h insulin AUC, which was ~43% lower than LF-HGI and HF-HGI (*p* < 0.001), and 28% lower than LF-LGI (*p* = 0.02). We conclude that in healthy adults, a meal with low GI and high in cereal fiber enhances postprandial endothelial function. Although the effect of a low-GI meal on reducing postprandial glucose AUC was independent of fiber, the effect of a low-GI meal on reducing postprandial insulin AUC was augmented by cereal fiber.

## 1. Introduction

Postprandial hyperglycemia is a risk factor for cardiovascular disease (CVD) even among apparently healthy adults without diabetes [1,2], and is a better predictor of mortality from all causes and CVD than is fasting blood glucose [3]. The increased risk for CVD associated with hyperglycemia may be due in part to the impairment in endothelial function associated with hyperglycemia [4,5]. Endothelial dysfunction is considered an important early indicator of atherogenic potential and precedes the development of atherosclerotic plaques [6,7]. Brachial artery flow-mediated dilation (FMD) is the most widely used non-invasive technique to assess endothelial function in humans [8], and a meta-analysis demonstrated that impairment in brachial artery FMD is significantly associated with future cardiovascular morbidity and mortality [9].

Most of the evidence demonstrating hyperglycemia-associated impairment in FMD comes from studies using oral glucose tolerance tests [4]. Such tests may not adequately reflect postprandial glucose responses following consumption of a mixed meal. The glycemic properties of a meal are influenced by a number of factors, including the glycemic index (GI) of the carbohydrates ingested [10,11], protein and fat content of the meal [12], and fiber content of the meal [13,14,15]. A recent meta-analysis indicated that meal ingestion generally reduces postprandial FMD [16], but the authors acknowledged that meal composition was not well described across studies. They recommended that future studies consider reporting the GI and fiber content of the meals.

In this regard, the study of Lavi et al. [15] is noteworthy. They reported that a high-GI breakfast meal that consisted of cornflakes significantly impaired postprandial FMD whereas a low-GI, breakfast meal containing a high-fiber cereal did not. However, the amount of fiber, macronutrient composition, and total energy content of the meals were not reported. Thus, the specific effects of GI and fiber on postprandial glycemia and FMD could not be ascertained. If the meals differed in protein and fat content, this could also have influenced the results [12]. Moreover, postprandial FMD was only measured for 2 h. We have previously reported that a high-carbohydrate, high-fiber breakfast meal significantly enhanced postprandial FMD at 4 h [17].

Consequently, significant gaps in the literature exist with respect to the impact of meals on postprandial FMD [4,16]. The purpose of this study was to assess the impact of both GI and dietary fiber (predominantly cereal fiber) on postprandial FMD in the setting of a typical breakfast meal. Healthy adults consumed four different meals on separate days and in randomized fashion. They consisted of a high-fiber, low-GI meal; a high-fiber, high-GI meal; a low-fiber, low-GI meal; and a low-fiber, high-GI meal. The difference in fiber content of the meals was due to cereal fiber. Importantly, meals were prepared so that they were similar in total energy and macronutrient composition. We hypothesized that (1) the meals high in cereal fiber, regardless of GI, would improve postprandial FMD, (2) the low-fiber, low-GI meal would have no effect of FMD, and (3) the low-fiber, high-GI meal would impair FMD.

## 2. Materials and Methods

### 2.1. Participants

Eleven adults participated in this study (mean ± SD age = 42.4 ± 16.1 years; weight = 70.5 ± 10.7 kg; BMI = 23.5 ± 3.1 kg/m^2^; percent fat = 26.0% ± 8.9%). Recruitment was via poster advertisement and word of mouth. Exclusion criteria were age less than 18 or greater than 64, smoking, food allergies, currently dieting to lose weight, pregnancy, current oral contraceptive usage, known cardiovascular or metabolic diseases, currently using anti-hypertensive, lipid or blood glucose lowering agents, current use of nutritional supplements other than daily multivitamins. The protocol was approved by the Institutional Review Board at the University of Virginia and was carried out in accordance with the Declaration of Helsinki. All participants provided written consent to participate.

### 2.2. Study Design

Each participant reported to the General Clinical Research Center (GCRC) between 7 AM and 9 AM on four occasions in a 12-h postabsorptive state (nothing but water after ~7 PM the night before). On each occasion participants consumed one of four, randomly assigned, meals. Before and at hourly intervals for 4 h after consuming each meal, blood was drawn from an indwelling venous catheter. Brachial artery FMD was performed before the meal and at 2 h and 4 h postprandial. These procedures are described below.

### 2.3. Meals

High-Fiber, Low-Glycemic Index (HF-LGI): The HF-LGI meal consisted of a high-fiber cereal (Kellogg’s All-Bran Buds) (36 g), enriched white bread (20 g), margarine spread (19 g), eggs (44 g), nonfat milk (290 g), apple slices (175 g), and apple juice (260 g).

Low-Fiber, Low-Glycemic Index (LF-LGI): The LF-LGI meal consisted of oatmeal cereal (40 g), enriched white bread (35 g), margarine spread (16 g), eggs (44 g), nonfat milk (275 g), and apple juice (285 g).

Low-Fiber, High-Glycemic Index (LF-HGI): The LF-HGI meal consisted of a low-fiber cereal (General Mills Golden Grahams) (36 g), plain bagel (110 g), margarine spread (18 g), eggs (44 g), and nonfat milk (75 g).

High-Fiber, High-Glycemic Index (HF-HGI): The HF-HGI meal consisted of a high-fiber cereal (Kellogg’s All-Bran Buds) (42 g), a high-GI cereal (General Mills Rice Chex) (30 g), plain bagel (60 g), margarine spread (17 g), eggs (44 g), nonfat milk (120 g), and seedless raisins (15 g).

All meals were prepared in the GCRC metabolic kitchen under the direction of the registered dietitian. The total energy content, macronutrient composition, and fiber content of each meal were determined using ProNutra software (Viocare Technologies, Princeton, NJ. Glycemic index was determined using ESHA Food Processor SQL Software (ESHA Research, Salem, OR). These data are presented in Table 1. The total energy content (~600 kcal), protein content (~21 g), total fat content (~15 g), saturated fat content (~3.5 g), mono-unsaturated fat content (~5 g), and poly-unsaturated fat content (~5 g) were the same for each meal. Total carbohydrate content was higher for the two high-fiber meals, but digestible carbohydrate content was approximately 90 g for each meal. The low- and high-GI meals differed markedly in terms of GI (43–44 vs. 70–71). Sodium was added, as necessary, to make all meals equivalent in sodium content. This was deemed necessary because a high-sodium meal has been reported to reduce postprandial FMD by more than a low-sodium meal [18].

### 2.4. Measurements

#### 2.4.1. Anthropometrics and Body Composition

Each participant had their height and weight measured, and body fat was assessed via air-displacement plethysmography (BodPod, LMI, Concord, CA). Standard prediction equations were used for estimating thoracic lung volume based on age, gender, height, weight, and ethnicity of the participant.

#### 2.4.2. Flow-Mediated Dilation (FMD)

To assess endothelium-dependent function, FMD was performed with high-resolution 2D and Doppler ultrasound (HDI 5000, ATL Philips Ultrasound, Andover, MA) using a linear-array transducer at a transmit frequency pf 12 MHz, as previously described [17,19]. Upon arrival to the GCRC, between 7 AM and 9 AM, participants rested in a supine position for 30 min in a quiet, dimly lighted, temperature-controlled room (22 to 23 °C). After the rest period, patients extended their non-dominant arm, which was immobilized by supports at approximately 75 to 80 degrees from the torso. Heart rate was continuously monitored by a three-lead electrocardiogram and triggers were applied to capture digital still images at the onset of the Q-wave (end diastole). Three baseline images were captured after a segment with a clear anterior and posterior intimal interface between the lumen and vessel wall was identified for continuous 2D gray-scale imaging. Then, a rapid inflation/deflation blood pressure cuff was placed 2 cm distal of the antecubital fold and inflated to 50 mm Hg above systolic blood pressure for 5 min. After five minutes, the cuff was rapidly deflated and digital still images were captured every 5 s from 30 s to 120 s post cuff release to determine peak dilation. The three highest consecutive peak values were used to determine the average dilation.

We followed the criteria set forth by the International Brachial Artery Reactivity Task Force [20]. All imaging was performed using the same sonographer who was blinded to the treatment condition. Image depth was initially set at 4 cm and gain settings were adjusted to provide an optimal view of the anterior and posterior arterial walls. Once optimal settings were obtained, they were kept constant throughout each meal condition. Images were taken in the longitudinal plane, proximal to the antecubital fold. The precise location of the ultrasound probe was individualized to optimize image clarity and avoid areas of arterial branching. All brachial artery images were captured and stored to an optical disk, and subsequently transferred to a dedicated PC outfitted with custom designed edge-detection and wall tracking software (Brachial Analyzer, Medical Imaging Applications, Iowa City, Iowa.). This software minimizes investigator bias and has a mean reported intra-observer coefficient of variation of 6.7% for repeated measures of FMD. In our lab, the intra-observer variability was 2.93%.

#### 2.4.3. Blood Analyses

Blood glucose (YSI 2700 Stat Plus, Yellow Springs, OH) and plasma insulin (Immulite 2000, Diagnostic Products, Los Angeles, CA) were measured at the GCRC core laboratory.

#### 2.4.4. Statistical Analyses

##### 2-h and 4-h Changes in FMD

2-h and 4-h changes in FMD were analyzed via linear mixed-effects analysis of covariance (ANCOVA). Dietary fiber, GI, time, fiber × GI interaction, fiber × time interaction, time × GI interaction, and fiber × GI × time interaction were seven sources of response-variable variation that were examined via ANCOVA. The response-variable variability attributable to baseline heterogeneity in the underlying FMD was also separated out and considered in the ANCOVA as concomitant variable-induced, response-variable variability. Formal tests for between-meal comparisons of 2-h and 4-h FMD were conducted by testing the null hypothesis that mean FMD is the same irrespective of the meal consumed. Null hypothesis tests were rejected based on a two-sided *p* < 0.05 criteria and both unadjusted *p*-values and Bonferroni-corrected *p*-values are reported for all between-meal comparisons.

##### Postprandial 1-h Blood Glucose and Plasma Insulin

Postprandial 1-h blood glucose and plasma insulin changes were analyzed via linear mixed-effects analysis of covariance (ANCOVA). The baseline and 1-h postprandial blood glucose and plasma insulin measurements were rescaled to the natural logarithmic scale and then the baseline rescaled measurements were subtracted from the 1-h postprandial rescaled measurements to produce the set of delta values that were utilized as the response-variable in the ANCOVA. Dietary fiber, GI, and fiber × GI interaction were three sources of response-variable variability that were examined via the ANCOVA. The response-variable variability attributable to baseline heterogeneity in the underlying response (e.g., baseline blood glucose) was also separated out and considered in the ANCOVA as concomitant variable-induced, response-variable variability. With regard to hypothesis testing, within-meal hypothesis testing was conducted by testing the null hypothesis that the mean change in log_e_ (response) is equal to zero. A two-sided *p* < 0.05 decision rule served as the null hypothesis rejection criterion for this test. Formal hypothesis testing for between-meal comparisons of the 1-h changes in blood glucose and plasma insulin were conducted by testing the null hypothesis that the mean 1-h change in the log_e_ (response) is the same irrespective of the meal consumed. This null hypothesis was rejected based on a two-sided *p* < 0.05 decision rule and both comparison-wise and multiple-comparison Bonferroni-corrected *p*-values are reported.

##### Glucose and Insulin Area under the Curve (AUC)

Area under curve (AUC) for blood glucose and plasma insulin was calculated by the trapezoidal rule. AUC measurements were then rescaled to the natural logarithmic scale and analyzed via linear mixed effects analysis of variance (ANOVA). Dietary fiber, GI, and fiber × GI interaction were the three sources of response-variable variability that were examined via the ANOVA. Formal tests for between-meal comparisons of blood glucose and plasma insulin AUC were conducted by testing the null hypothesis that the log_e_ (AUC) is the same irrespective of the meal consumed. This null hypothesis was rejected based on a two-sided *p* < 0.05 decision rule and both comparison-wise and multiple-comparison Bonferroni-corrected *p*-values are reported.

## 3. Results

### 3.1. Flow-Mediated Dilation

**Figure 1:**
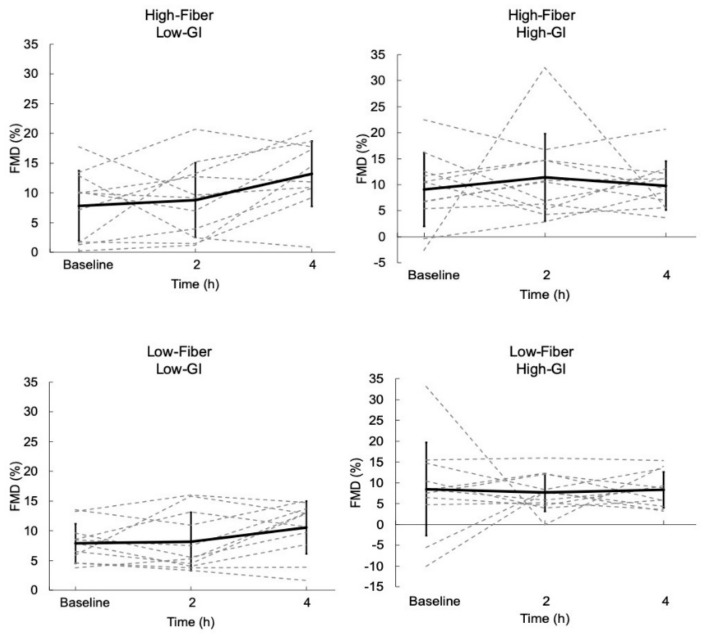
Postprandial change in flow-mediated dilation (FMD) across 4 h for each meal. Dashed lines represent responses for each individual. Solid lines represent mean responses (± SD). FMD was not different across time points for all meals except High-Fiber, Low-GI at 4 h compared to baseline (*p* = 0.021; Bonferroni adjusted *p* = 0.165).

The FMD results are displayed in Figure 1. FMD was increased 4 h after the HF-LGI meal, from 7.8% ± 5.9% to 13.2% ± 5.5% (*p* = 0.021); Bonferroni adjusted (*p* = 0.165), but was unchanged at 2 h. FMD was unchanged at 2 h and 4 h after the other three meals (Figure 1).

### 3.2. Blood Glucose

**Figure 2:**
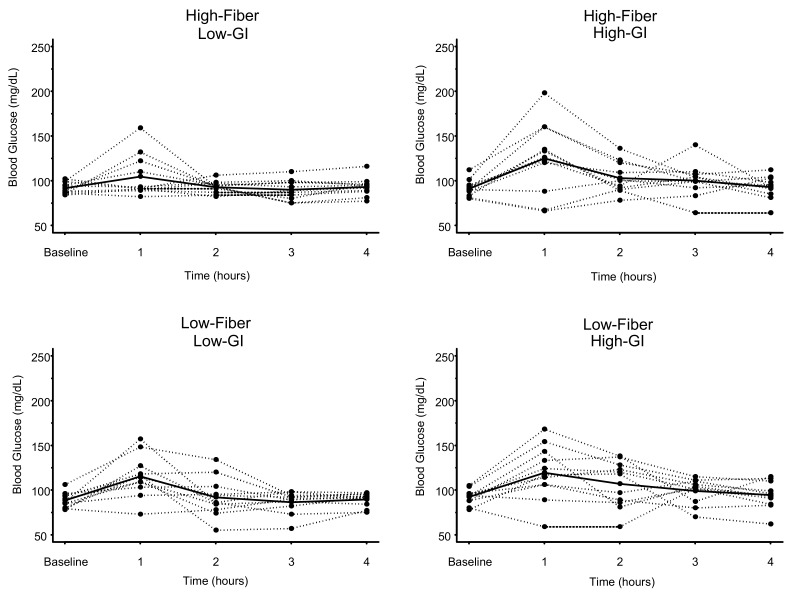
Glucose concentrations over time in response to four meals differing in glycemic index (GI) and fiber content. Dashed lines represent individual subject responses. Solid lines represent mean responses for all subjects. See text and Table 2 for statistical comparisons.

**Figure 3:**
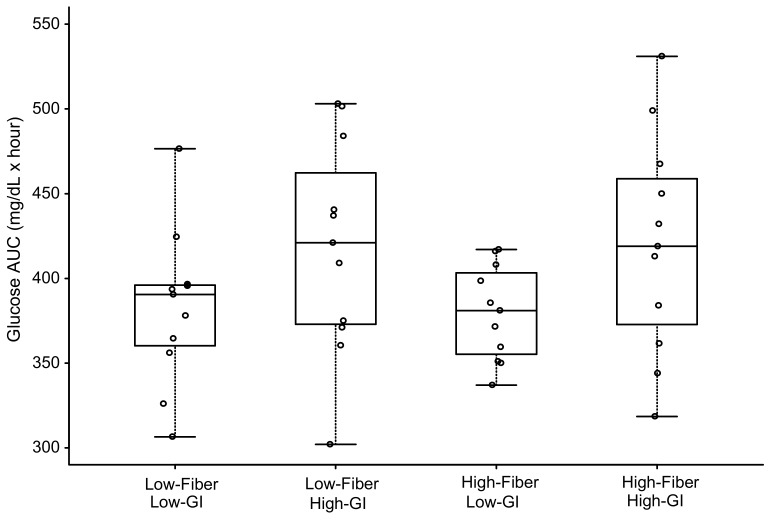
Glycemic response as area under curve (AUC) over 4 h in response to four meals differing in glycemic index (GI) and fiber content. Boxes for each meal represent the interquartile range. Minimum and maximum values are indicated at the tips of each vertical line. The median for each meal is depicted by the horizontal line within each box. See Table 3 for statistical comparisons between meals.

The individual and mean glucose responses to each meal are displayed in Figure 2. Blood glucose at 1 h postprandial was significantly elevated by ~28–31% for all meals (*p* range 0.001 to 0.005) except the HF-LGI meal (*p* = 0.13) (Table 2). For glucose AUC, there was a significant effect for GI (*p* = 0.001) but not for either fiber or GI × fiber interaction (Figure 3 and Table 3). The two low-GI meals had significantly lower (~9%) glucose 4-h AUC than the two high-GI meals, regardless of fiber content (Table 3). Dietary fiber had no effect on reducing the glucose AUC of the high-GI meals (420 ± 65 mg/dL × hour vs. 419 ± 64 mg/dL × hour) (Table 3).

### 3.3. Plasma Insulin

**Figure 4:**
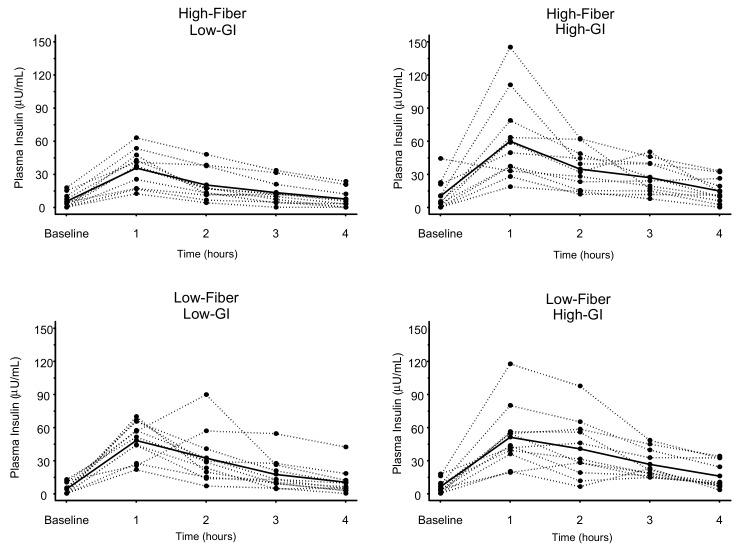
Insulin responses to four meals differing in glycemic index (GI) and dietary fiber content. Dashed lines represent individual subject responses. Solid lines represent mean responses for all subjects. See text and Table 4 for statistical comparisons between meals.

**Figure 5:**
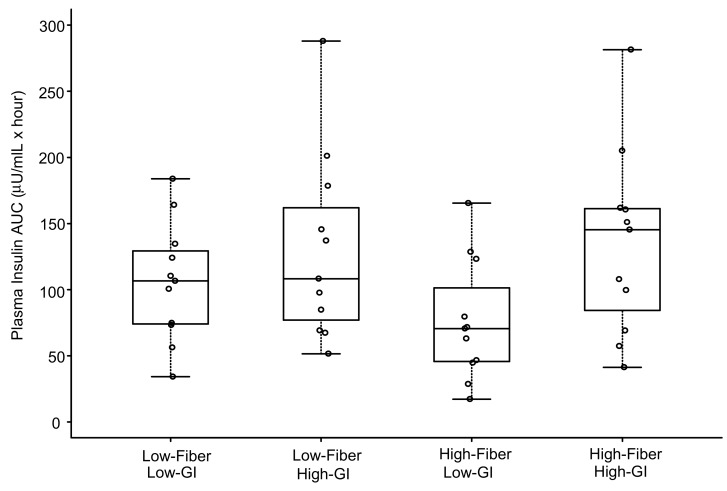
Plasma insulin area under curve (AUC) over 4 h in response to four meals differing in glycemic index (GI) and fiber content. Boxes for each meal represent the interquartile range. Minimum and maximum values are indicated at the tips of each vertical line. The median for each meal is depicted by the horizontal line within each box. See Table 5 for statistical comparisons between meals.

The individual and mean plasma insulin responses to each meal are displayed in Figure 4. Similar to the results for glucose, 1-h plasma insulin (Table 4) was significantly elevated above baseline concentrations for all meals (*p* < 0.003) except the HF-LGI meal (*p* = 0.11). For insulin AUC, both LGI meals (HF-LGI = 76 ± 46 µU/mL × hour; LF-LGI = 106 ± 45 µU/mL × hour) had significantly lower values compared to both HGI meals (HF-HGI = 135 ± 70 µU/mL × hour; LF-HGI = 130 ± 71 µU/mL × hour), and the HF-LGI meal had a significantly lower insulin AUC compared to the LF-LGI meal (Figure 5 and Table 5). Thus, the maximum attenuation of insulin AUC was due to the combination of high fiber and low GI.

## 4. Discussion

The major novel finding of the present study is that FMD was enhanced after the meal that was both high in dietary fiber and low in GI. This result is consistent with our previous report showing that a high-carbohydrate, high-fiber meal significantly improved FMD in adults with the metabolic syndrome [17]. We did not address the effect of GI in that study, although the dominant source of dietary fiber was the same (a wheat bran cereal). Subsequent calculation indicated that the GI of the high-carbohydrate, high-fiber meal we used in our previous study was approximately 56. We also did not assess postprandial glucose and insulin responses in that study. The combination of both low GI and high cereal fiber may be crucial because postprandial FMD was increased only after the HF-LGI meal but was not changed after the LF-LGI meal or the HF-HGI meal. Thus, our hypothesis regarding the beneficial impact of cereal fiber on FMD regardless of GI is rejected.

Endothelial function is typically impaired after ingestion of a meal, although considerable heterogeneity in postprandial FMD responses is evident [16]. We hypothesized that FMD would be impaired after the LF-HGI meal. The lack of a decrease in FMD after this meal, or after the LF-LGI meal, which we hypothesized would result in no impairment due to the low GI of the meal, may be due to several factors. Postprandial FMD impairment is less likely to be observed in individuals with baseline FMD < 10% and in individuals with normal fasting glucose [16]. Our meals were low in fat (~15 g), and thus would not be expected to result in lipemia-induced endothelial dysfunction [21]. Moreover, most of the studies showing FMD impairment associated with hyperglycemia have used oral glucose loading [4]. Oral glucose tolerance tests (typically 75 g) result in greater postprandial glucose increases compared to mixed meals [22]. Even with ~90 grams of digestible carbohydrate, our meals only increased blood glucose by ~30–40% at 1 h postprandial, with 1-h glucose concentrations ranging from 105 ± 24 mg/dL for HF-LGI to 125 ± 40 mg/dL for HF/HGI. Such minor elevations in postprandial glucose are generally not associated with impairment in FMD [23]. Finally, inclusion of protein and fat in meals attenuates the postprandial rise in glucose [12,24]. Thus, all of these factors may have contributed to lack of FMD decrement in the present study.

Ours is the first study to distinguish the separate effects of GI and dietary fiber on postprandial FMD, glucose and insulin. Lavi et al. [15] previously reported that a breakfast meal consisting of a high-fiber cereal with an estimated GI of 40 prevented a reduction in FMD that was observed after ingestion of a breakfast meal consisting of a low-fiber cereal with an estimated GI of 80. Compared to our study, their experimental design differed in several respects. First, postprandial FMD was measured only at 2 h. Our data show that the beneficial effect of a high-fiber, low-GI meal may not be evident until 4 h postprandial, and this may be due to the delayed and/or prolonged antioxidant effects of cereal fiber during the postprandial period (see discussion below). Second, the source and amount of fiber in their high-fiber cereal were not reported. Moreover, macronutrient and total energy content of their meals were not reported. Lavi et al. [15] concluded that a low-GI meal was key in preventing postprandial impairment in FMD. However, their low-GI meal was also high in cereal fiber. Thus, the separate effects of cereal fiber and GI could not be determined. Our results demonstrate that a low-GI meal that is low in cereal fiber does not have the same beneficial effect on FMD as does a low-GI meal with a high amount of cereal fiber.

The high-fiber cereal we used in the present study may be relevant to explaining our results. Wheat bran, the major ingredient in the cereal, is predominantly insoluble fiber [25]. Insoluble fiber intake is generally more strongly associated with reduced CVD and coronary heart disease than either soluble fiber or total fiber [26]. Cereal fiber contains considerable amounts of antioxidants, which may reduce inflammation and could conceivably have a beneficial impact on endothelial function in the postprandial period [27,28,29,30]. The antioxidant capacity of wheat bran is especially high [30]. Plasma concentrations of phytophenols in wheat bran peak within the first 1–2 h post ingestion of wheat bran-rich cereals, and they stay elevated above baseline levels for 5–6 h [27,28]. This may explain why we did not observe a significantly elevated FMD after the HF-LGI meal until 4 h postprandial. However, it is also important to acknowledge the influence of GI because the HF-HGI meal did not enhance postprandial FMD at any time point. This demonstrates that a high-fiber breakfast meal only enhances postprandial FMD when it has a low GI.

The insulin response to oral glucose loading has been shown to be a significant predictor of coronary artery disease in non-diabetic adults [31,32]. The HF-LGI meal elicited the lowest insulin AUC and, similar to the results for glucose, was the only meal for which 1-h insulin was not significantly different from baseline. It has previously been reported that insulin impairs endothelial function [22,33,34], although this finding has not been observed in all studies [35]. However, direct comparison of results from these studies is complicated by the fact that most of them used insulin infusion [33,34,35] rather than a true meal ingestion. The reduction in FMD after oral glucose loading [22,36] and meal ingestion [22] has been reported to be significantly inversely correlated to the insulin AUC. To examine this association in the current study, we performed regression analyses on the relationship between 4-h insulin AUC and the change in 4-h FMD for each meal. None of the regression analyses produced a slope significantly different from zero (all *p*-values > 0.24). Thus, our results suggest that the FMD response following our HF-LGI meal is not attributable to the attenuated postprandial insulin response to that meal.

Postprandial glycemia is a significant risk factor for CVD [1,2,3]. Breakfast meals containing 13–26 g cereal fiber significantly attenuate postprandial glucose responses [13,14]. In our study, 1-h glucose was elevated above baseline for all meals except HF-LGI. But fiber had no effect on 4-h glucose AUC because both low-GI meals had similar 4-h glucose AUCs, regardless of fiber content, and both high-GI meals had similar glucose AUCs regardless of fiber content. The glucose AUCs, however, were ~9% lower following the two low-GI meals. Thus, the glucose AUC data indicate that the blood glucose-lowering effect of low-GI meals is primarily due to the glycemic properties of the foods rather than the fiber content. Although fiber-rich foods generally have a low-GI, not all low-GI foods have a high fiber content [37].

Although the present study focused on the immediate postprandial period, our data may have implications for CVD risk reduction. In addition to the documented CVD risk associated with postprandial glycemia [1,2,3], observational studies also have reported that dietary GI is associated with increased risk of CVD [2]. Prospective cohort studies consistently show reduced CVD risk associated with cereal fiber intake [38]. A recent meta-analysis indicated that CVD mortality risk was reduced by 18% when comparing the highest vs. lowest consumption of cereal fiber [39]. A pooled analysis of cohort studies indicated that each 10 g/day increase in cereal fiber intake was associated with a 25% lower risk of coronary heart disease mortality [40]. Dietary GI was not considered in these meta-analyses. Although GI was the primary determinant of postprandial glucose responses in our study, only the low-GI meal that was high in cereal fiber enhanced postprandial FMD. This may be important because impairment in FMD is a significant predictor of future cardiovascular morbidity and mortality [9], and it is likely that most adults in Western populations spend much of their day and evening in a postprandial state. Because not all low-GI foods have a high fiber content [37], it is possible to have a low-GI diet that is also low in dietary fiber. Such low-GI diets might not be as healthy as a similarly low-GI diet that is high in fiber. In this regard it is worth noting that in a 13-yr prospective study of 2897 older adults, a diet that was both high in GI and low in cereal fiber was associated with greater risk (relative risk, RR = 5.06) of stroke compared to diets with either high GI (RR = 1.91) or low cereal fiber intake (RR = 2.13) alone [41].

### Strengths and Limitations

Our study has several strengths. We had rigorous control of all four meals, with total energy and macronutrient composition and sodium content the same for each meal. The meals differed only in GI and fiber content. To our knowledge, this is the only study with this design. We also measured FMD up to 4 h postprandial, which may provide better insight into the effects of meal ingestion on endothelial function. As noted in a recent meta-analysis, most previous studies have measured FMD 1–3 h postprandial [16]. We also contend that by studying postprandial responses to meals that contained all macronutrients, our results are more translatable to the general population. The majority of studies have not provided details on meal composition, fiber content, GI, or total energy [16].

Our study also has some limitations. We did not measure markers of oxidative stress, antioxidant status, or levels of cereal fiber compounds that could have impacted FMD. Although the amount of protein and fat did not differ across meals (Table 1), because the sources of protein and fat for each meal were not identical, it is possible that the amount of bioactive peptides and fatty acids could have differed across meals. Our earliest postprandial blood sample was at 1 h, thus we may have missed earlier glucose and insulin peaks. However, several studies have shown similar concentrations of glucose and insulin at 30 and 60 min after an OGTT or meal ingestion [15,24,42,43]. Our sample size was relatively small, and a number of our statistical comparisons were only significant with unadjusted *p*-values. Thus, our results need to be confirmed with additional studies with larger sample sizes. For our primary outcome, FMD, we used the mean and SD data on Δ 4-h FMD for the three meals that had no change in postprandial FMD to estimate the sample size required to have 0.80 statistical power to reject the null hypothesis that the mean 4-h Δ FMD = 0. For the Low-Fiber, Low-GI meal (4-h Δ FMD = 2.7% ± 3.8%), 16 subjects would be needed. By contrast, >300 subjects would be needed for the High-Fiber, High-GI meal (Δ 4-h FMD = 0.8% ± 4.8%) and the Low-Fiber, High-GI meal (Δ 4-h FMD = −0.1% ± 8.3%). Our results demonstrate that only low-GI meals are likely to have a positive impact on postprandial FMD in adults. It was not our intent to examine sex differences, and we were not powered to do so. A meta-analysis indicated that women are less likely than men to experience FMD impairment after meal ingestion [16]. Although FMD was not impaired after any of our meals, we were interested in knowing whether there might be a sex difference in the HF-LGI condition in which FMD was increased. In the five men, baseline to 4-h postprandial FMD was increased from 5.6% ± 5.7% to 13.8% ± 4.2%; in the six women, FMD was increased from 9.7% ± 6.3% to 12.7% ± 3.9%. These were not statistically different from one another, suggesting no sex difference in response to the HF-LGI meal.

## 5. Conclusions

In healthy men and women, consumption of a breakfast meal that is high in cereal fiber (20 g; 16–18 g of wheat bran) and low in GI significantly improved FMD 4 h after ingestion. Our data show that it is the combination of high cereal fiber and low GI that produced this beneficial effect on postprandial FMD, whereas a low-GI meal that is low in cereal fiber, and a high-GI meal that is high in cereal fiber, did not. Our results also demonstrate that GI is more important than fiber for attenuating the postprandial glucose response, but the greatest reduction in the postprandial insulin response occurred when the low-GI meal was also high in fiber. Collectively, our data suggest that the full benefit of a low-GI meal for minimizing postprandial dysmetabolism may depend in large part on cereal fiber. FMD is significantly associated with future cardiovascular events [9] and postprandial hyperglycemia and hyperinsulinemia have been shown to be associated with CVD [1,2,3,31,32]. Because most adults in Western populations spend the majority of their day in a postprandial state, these results may have clinical utility for nutritional counseling.

## Figures and Tables

**Table 1:** Nutrient composition and glycemic index of the study meals.

Nutrient	High-Fiber Low-GI	Low-Fiber Low-GI	Low-Fiber High-GI	High-Fiber High-GI
Glycemic Index	44	43	70	71
Total Fiber (g)	20.4	4.3	3.6	20.3
Total Kcal	600	601	601	606
Total Protein (g)	20.0	20.9	21.5	21.1
Total Carbohydrate (g)	108.3	96.3	92.8	109.8
Total Fat (g)	15.7	15.6	15.8	14.9
Saturated Fat (g)	3.6	3.6	3.6	3.4
Monounsaturated Fat (g)	5.2	5.3	5.2	4.9
Polyunsaturated Fat (g)	5.4	5.0	5.4	5.1
Trans Fat (g)	0.9	0.7	0.8	0.8
Sodium (mg)	1007	1033	1039	1011

**Table 2:** Blood glucose responses 1-h postprandial: Comparisons between meals.

Meal	Glucose mg/dL (mean ± SD)		High-Fiber Low-GI	Low-Fiber Low-GI	High-Fiber High-GI	Low-Fiber High-GI
	Baseline	1-h	Unadjusted *p*-Values (Bonferroni-Adjusted *p*-Values)			
High-Fiber Low-GI	91.6 ± 6.4	104.5 ± 23.9	-	0.025 (0.150)	0.018 (0.108)	0.086 (0.516)
Low-Fiber Low-GI	88.4 ± 8.5	115.3 ± 23.3 *		-	0.933 (1.000)	0.532 (1.000)
High-Fiber High-GI	91.1 ± 9.3	125.0 ± 40.2 *			-	0.464 (1.000)
Low-Fiber High-GI	92.3 ± 8.5	119.4 ± 30.5 *				-

GI = Glycemic Index; SD = Standard deviation; *, Significantly higher than baseline blood glucose for that meal (*p* < 0.005).

**Table 3:** Glucose area under curve: Comparisons between meals.

Meal	Glucose AUC mg/dL × hour (mean ± SD)	High-Fiber Low-GI	Low-Fiber Low-GI	High-Fiber High-GI	Low-Fiber High-GI
		Unadjusted *p*-Values (Bonferroni-Adjusted *p*-Values)			
High-Fiber Low-GI	380 ± 28	-	0.916 (1.000)	0.015 (0.090)	0.019 (0.114)
Low-Fiber Low-GI	383 ± 46		-	0.020 (0.120)	0.024 (0.144)
High-Fiber High-GI	420 ± 65			-	0.933 (1.000)
Low-Fiber High-GI	419 ± 64				-

AUC = Area Under Curve for entire 4-h postprandial period; GI = Glycemic index; SD = Standard deviation.

**Table 4:** Plasma insulin responses 1-h postprandial: Comparisons between meals.

Meal	Insulin µU/mL (mean ± SD)		High-Fiber Low-GI	Low-Fiber Low-GI	High-Fiber High-GI	Low-Fiber High-GI
	Baseline	1-h	Unadjusted *p*-Values (Bonferroni-Adjusted *p*-Values)			
High-Fiber Low-GI	5.5 ± 6.5	35.8 ± 16.2	-	0.032 (0.192)	0.011 (0.066)	0.001 (0.006)
Low-Fiber Low-GI	4.2 ± 5.1	48.3 ± 17.2 *		-	0.621 (1.000)	0.169 (1.000)
High-Fiber High-GI	10.2 ± 13.9	60.1 ± 38.5 *			-	0.353 (1.000)
Low-Fiber High-GI	6.3 ± 6.2	51.2 ± 27.9 *				-

GI = Glycemic index; SD = Standard deviation; *, Significantly higher than baseline blood glucose for that meal (*p* ≤ 0.003). See text for details on statistical analysis for differences between meals.

**Table 5:** Insulin area under curve: Comparisons between meals.

Meal	Insulin AUC µU/mL × hour (mean ± SD)	High-Fiber Low-GI	Low-Fiber Low-GI	High-Fiber High-GI	Low-Fiber High-GI
		Unadjusted *p*-Values (Bonferroni-Adjusted *p*-Values)			
High-Fiber Low-GI	76 ± 46	-	0.001 (0.006)	<0.001 (<0.006)	<0.001 (<0.006)
Low-Fiber Low-GI	106 ± 45		-	0.003 (0.018)	0.021 (0.126)
High-Fiber High-GI	135 ± 70			-	0.673 (1.000)
Low-Fiber High-GI	130 ± 71				-

AUC = Area Under Curve for entire 4-h postprandial period; GI = Glycemic index; SD = Standard deviation.
